# Supplementary material for: Gene Conversion Facilitates the Adaptive Evolution of Self-Resistance in Highly Toxic Newts
Source: Mol Biol Evol. 2021 Jun 15;38(10):4077–94. doi: 10.1093/molbev/msab182 (PMC8476164; doi:10.1093/molbev/msab182)
Supplement: msab182_Supplementary_Data [file msab182_supplementary_data.pdf]

Supporting Information

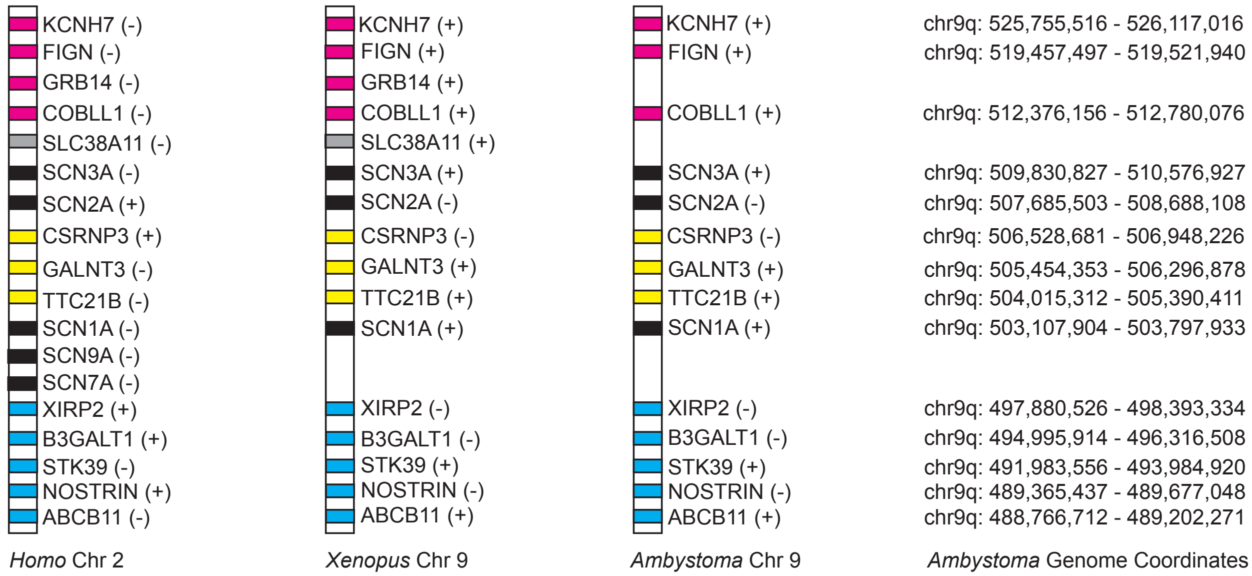

Fig. S1. Conserved synteny of voltage-gated sodium channel paralogs across tetrapods**.** The genetic configuration of brain/nerve voltage-gated sodium channels (*SCNA* genes) is highly conserved across three tetrapod species: humans (*Homo*), frogs (*Xenopus*), and salamanders (*Ambystoma*). Genome coordinates are based on the AmexG.v6 assembly. Symbols (+) and (-) refer to gene orientation within this reference genome.

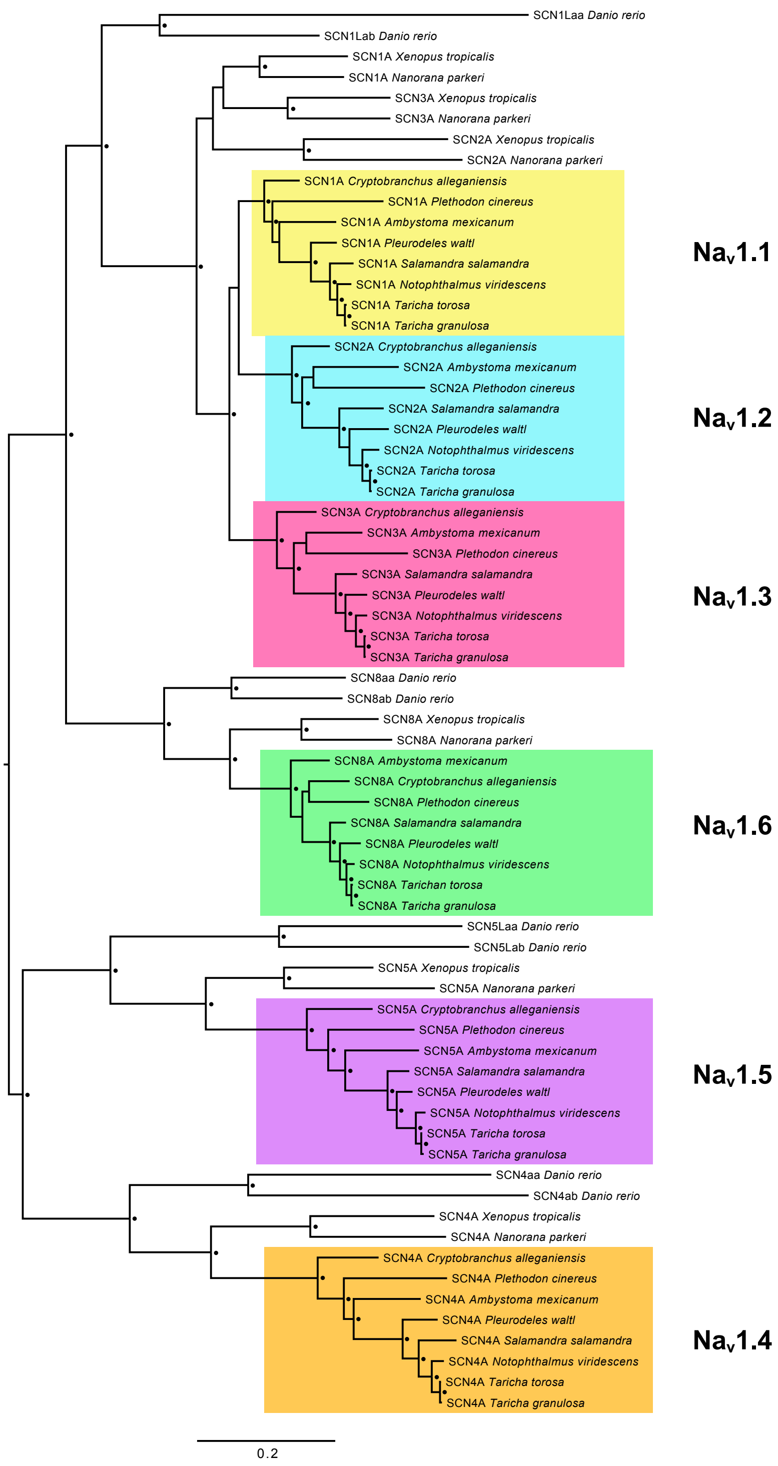

Fig. S2. Maximum likelihood tree constructed from 6789 bp coding sequence alignment of salamander Na_v genes with coding sequences from frogs (*Nanorana parkeri* and *Xenopus tropicalis*) and fish (*Danio rerio*) as outgroups. Black circles indicate nodes with bootstrap support >90%.

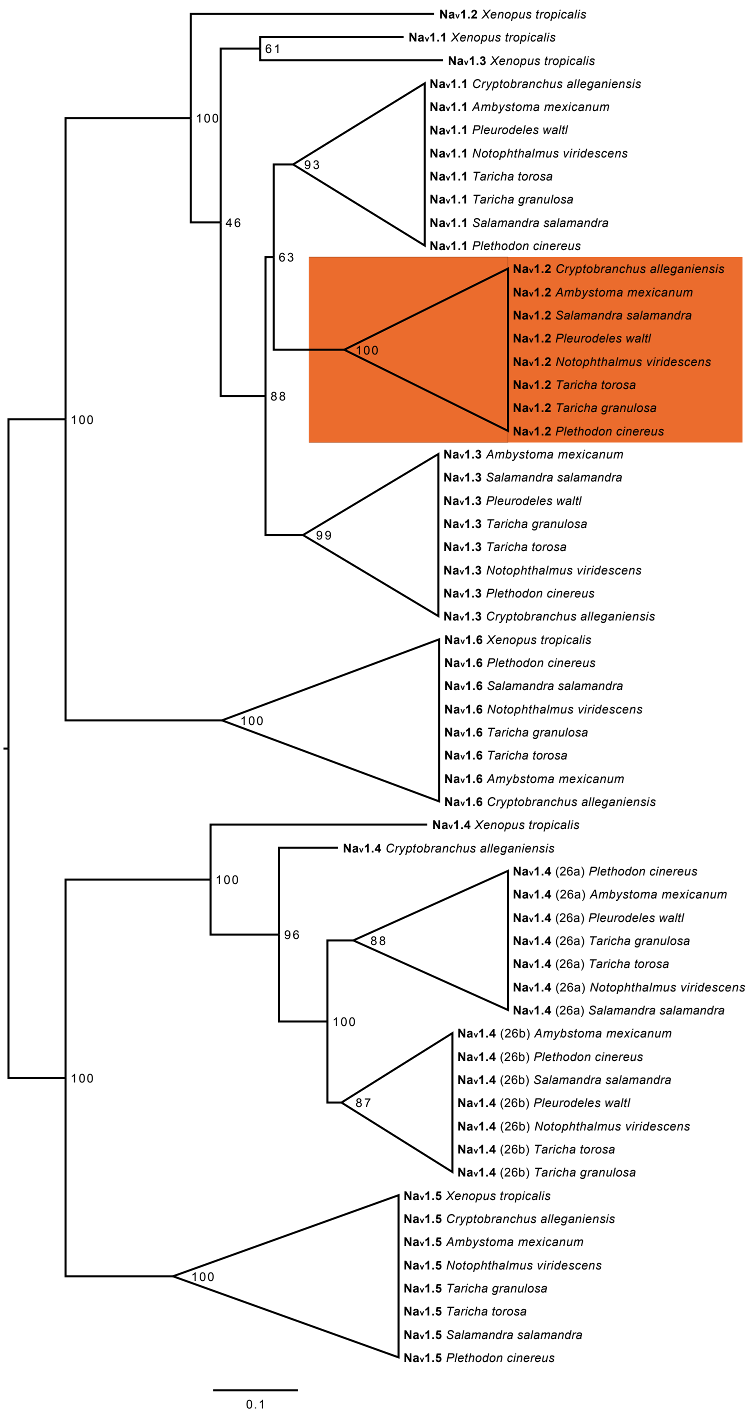

Fig. S3. Maximum likelihood tree constructed from 1063 bp nucleotide alignment of *SCNA* exon 26 sequences. Node labels indicate bootstrap support from 100 replicates. Orange highlighting indicates the clade grouping *SCN2A* from *Ambystoma* with *SCN2A* from other salamander species (bootstrap support 100%).

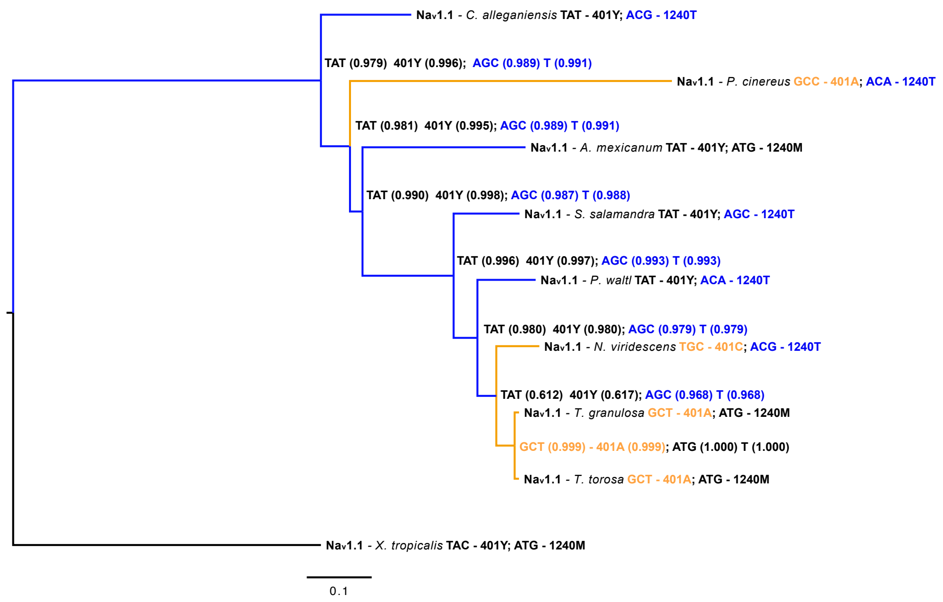

Fig. S4. Ancestral sequence reconstruction of tetrodotoxin resistant substitutions in $Na_v1.1$ based on neutral site model (M8a). Numbers in parentheses indicate posterior probability support for ancestral sequence reconstruction at nodes. Branch lengths indicate number of synonymous substitutions per codon. Blue branches – moderately resistant substitution, orange branches – highly resistant substitution.

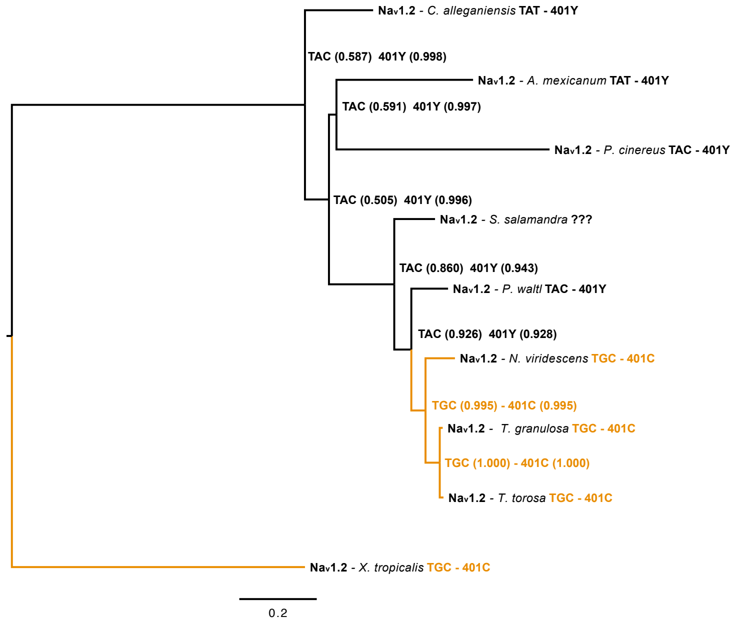

Fig. S5. Ancestral sequence reconstruction of tetrodotoxin resistant substitutions in $Na_v1.2$ based on neutral site model (M8a). Numbers in parentheses indicate posterior probability support for ancestral sequence reconstruction at nodes. Branch lengths indicate number of synonymous substitutions per codon. Blue branches – moderately resistant substitution, orange branches – highly resistant substitution.

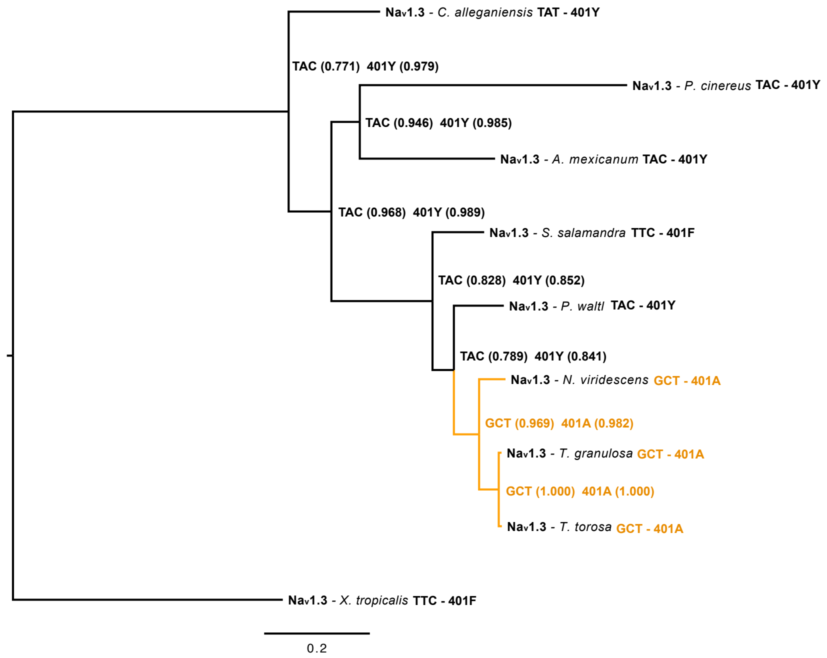

Fig. S6. Ancestral sequence reconstruction of tetrodotoxin resistant substitutions in $Na_v1.3$ based on site selection model (M8). Numbers in parentheses indicate posterior probability support for ancestral sequence reconstruction at nodes. Branch lengths indicate number of synonymous substitutions per codon. Blue branches – moderately resistant substitution, orange branches – highly resistant substitution.

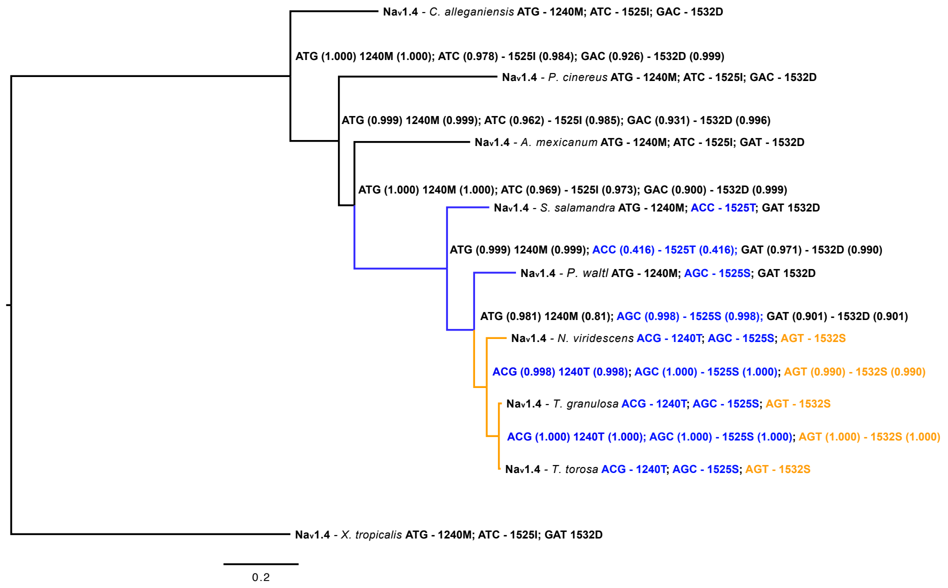

Fig. S7. Ancestral sequence reconstruction of tetrodotoxin resistant substitutions in $Na_v1.4$ based on neutral site model (M8a). Numbers in parentheses indicate posterior probability support for ancestral sequence reconstruction at nodes. Branch lengths indicate number of synonymous substitutions per codon. Blue branches – moderately resistant substitution, orange branches – highly resistant substitution.

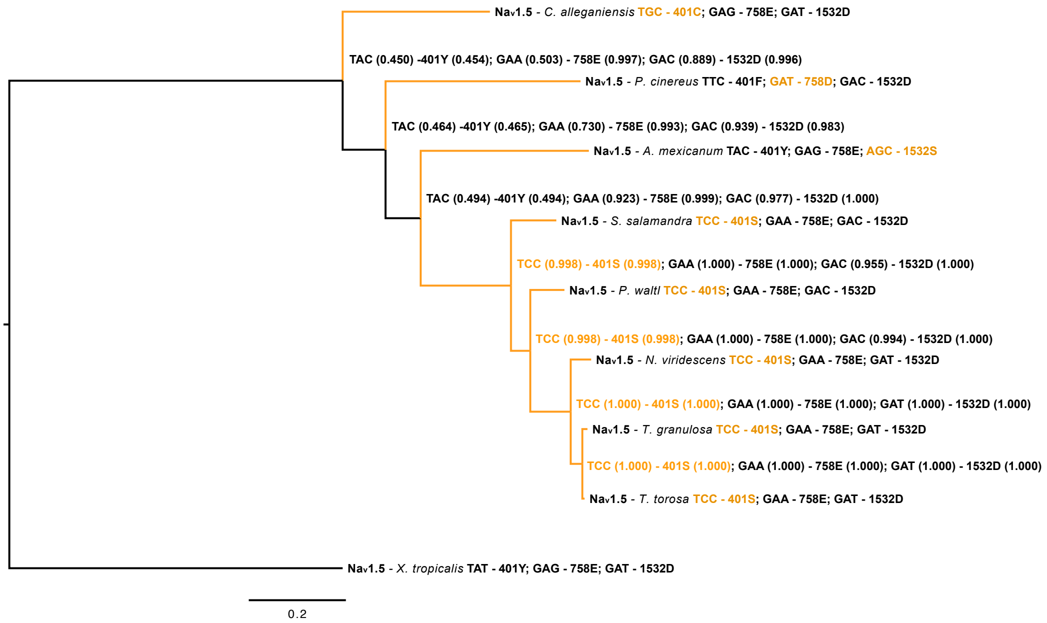

Fig. S8. Ancestral sequence reconstruction of tetrodotoxin resistant substitutions in Na$_v$1.5 based on neutral site model (M8a). Numbers in parentheses indicate posterior probability support for ancestral sequence reconstruction at nodes. Branch lengths indicate number of synonymous substitutions per codon. Blue branches – moderately resistant substitution, orange branches – highly resistant substitution.

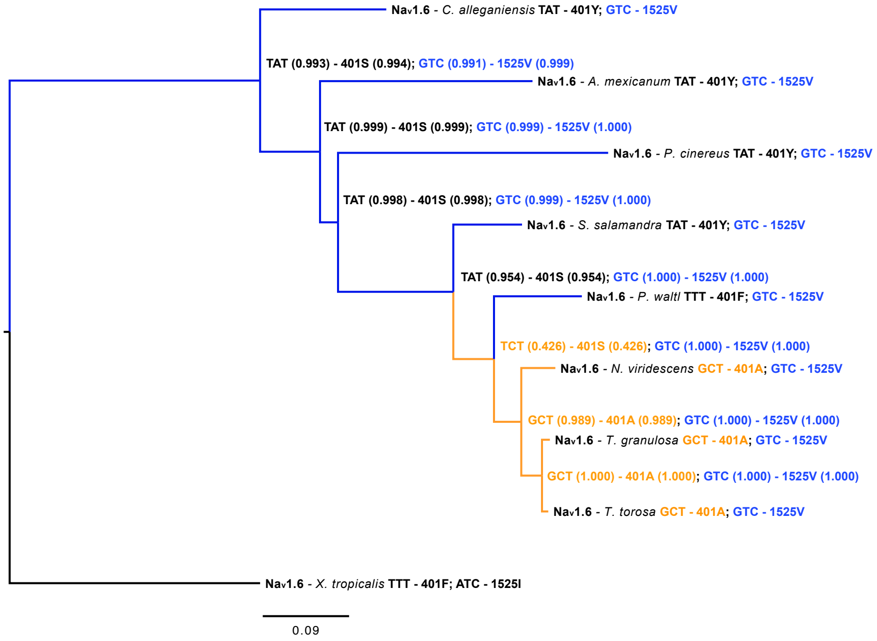

Fig. S9. Ancestral sequence reconstruction of tetrodotoxin resistant substitutions in Na$_v$1.6 based on neutral site model (M8a). Numbers in parentheses indicate posterior probability support for ancestral sequence reconstruction at nodes. Branch lengths indicate number of synonymous substitutions per codon. Blue branches – moderately resistant substitution, orange branches – highly resistant substitution.

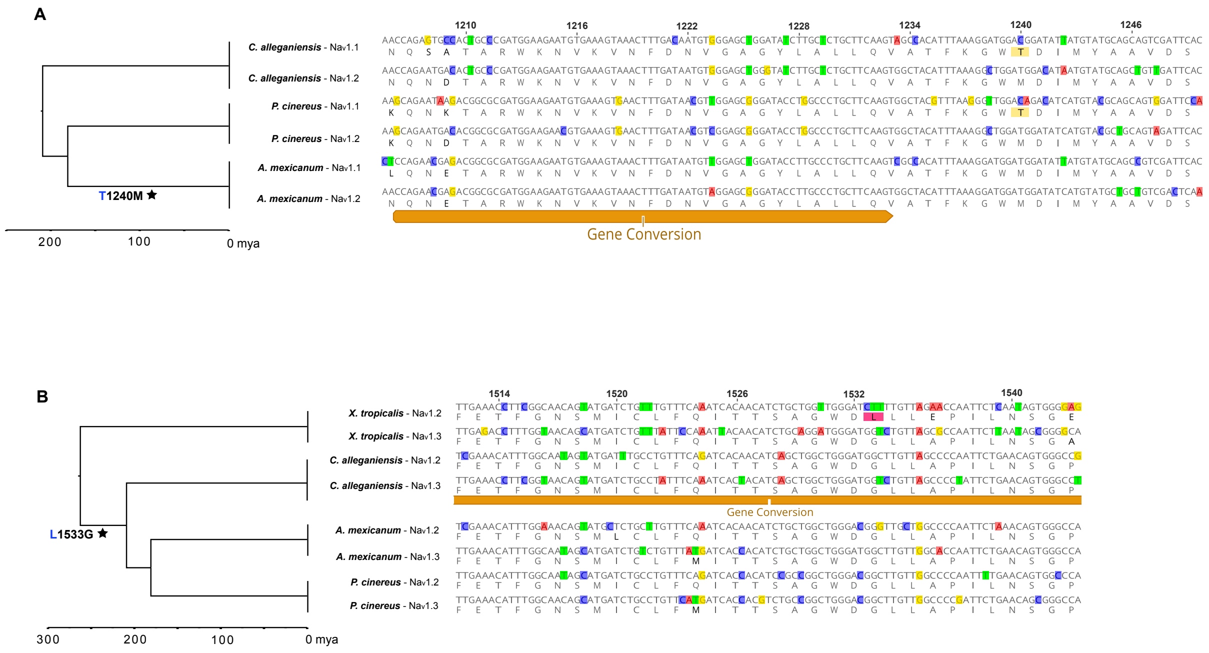

Fig. S10. Gene conversion within TTX binding regions associated with a loss of resistance-conferring substitutions. (A) Gene conversion was detected between the DIII P-loops of $Na_v1.1$ and $Na_v1.2$ in both *A. mexicanum*. The 1240T substitution conferring TTX resistance is highlighted in yellow. (B) Gene conversion was also detected between DIV P-loops of $Na_v1.2$ and $Na_v1.3$ within *C. alleganiensis*. The 1533L substitution putatively conferring TTX resistance is highlighted in pink. Site numbers are in reference to amino acids of rat $Na_v1.4$ (accession number AAA41682).

Table S1. Summary Statistics from Salamander Na_v Sequencing and Alignments

**SCN1A (Na_v1.1)**	**GC %**	**Length**	**Gaps**	**% Gaps**	**% Complete[a]**	**Pairwise % identity[b]**	**Average coverage**
Ambystoma mexicanum	42.3	6224	165	2.7	97.3	81.8	-
Pleurodeles waltl	42.0	6149	390	6.3	93.7	81.9	-
Cryptobranchus alleganiensis	42.5	6216	162	2.6	97.4	81.6	21.44
Notophthalmus viridescens	41.5	6188	231	3.7	96.3	84.3	21.33
Taricha granulosa	41.5	6224	201	3.2	96.8	85.2	42.41
Taricha torosa	41.4	6224	159	2.6	97.4	85.4	39.88
Salamandra salamandra	41.6	5915	1846	31.2	68.8	60.2	-
Plethodon cinereus	46.1	6219	477	7.7	92.3	75.1	13.42
Xenopus tropicalis	41.2	6216	153	2.5	97.5	73.4	-
SCN2A (Na_v1.2)	**GC %**	**Length**	**Gaps**	**% Gaps**	**% Complete**	**Pairwise % Identity**	**Average coverage**
Ambystoma mexicanum	43.5	5946	402	6.8	93.2	70.9	-
Pleurodeles waltl	41.5	5982	108	1.8	98.2	79.7	-
Cryptobranchus alleganiensis	41.3	6000	438	7.3	92.7	74.9	18.6
Notophthalmus viridescens	42.3	6039	432	7.2	92.8	13.4	22.54
Taricha granulosa	41.8	6060	75	1.2	98.8	81.7	40.43
Taricha torosa	41.9	6060	69	1.1	98.9	81.6	34.32
Salamandra salamandra	41.9	3672	1077	29.3	70.7	56.8	-
Plethodon cinereus	46.7	6006	700	10	90.0	68.4	22.7
Xenopus tropicalis	41.4	6054	141	2.3	97.7	66.4	-
SCN3A (Na_v1.3)	**GC %**	**Length**	**Gaps**	**% Gaps**	**% Complete**	**Pairwise % Identity**	**Average coverage**
Ambystoma mexicanum	45	6072	51	0.8	99.2	83.3	-
Pleurodeles waltl	42.3	6069	195	3.2	96.8	85.9	-
Cryptobranchus alleganiensis	43.3	6093	39	0.6	99.4	83.7	23.59
Notophthalmus viridescens	42.4	6078	72	1.2	98.8	88.3	49.48
Taricha granulosa	42.3	6093	72	1.2	98.8	88.6	42.71
Taricha torosa	42.2	6099	72	1.2	98.8	88.6	40.9
Salamandra salamandra	42.9	6096	186	3.1	96.9	85.3	-
Plethodon cinereus	47.9	6096	510	8.4	91.6	75.1	14.44
Xenopus tropicalis	42.2	6093	36	0.6	99.4	75.5	-
SCN4A (Na_v1.4)	**GC %**	**Length**	**Gaps**	**% Gaps**	**% Complete**	**Pairwise % Identity**	**Average coverage**
Ambystoma mexicanum	43.4	5034	141	2.8	97.2	81.8	-
Pleurodeles waltl	43.3	5622	96	1.7	98.3	85.3	-
Cryptobranchus alleganiensis	46.2	5580	48	0.9	99.1	80.9	12.46
Notophthalmus viridescens	43.6	5622	96	1.7	98.3	87.1	47.29
Taricha granulosa	44	5469	87	1.6	98.4	87.3	35.46
Taricha torosa	44	5469	90	1.6	98.4	87.4	42.97
Salamandra salamandra	44.5	5619	135	2.4	97.6	85.4	-
Plethodon cinereus	43.5	5628	279	5	95	78.2	12.2
Xenopus tropicalis	43.3	5622	111	2	98	71.2	-
SCN5A (Na_v1.5)	**GC %**	**Length**	**Gaps**	**% Gaps**	**% Complete**	**Pairwise % Identity**	**Average coverage**
Ambystoma mexicanum	48.9	5988	45	0.8	99.2	82.7	-
Pleurodeles waltl	44.7	5706	102	1.8	98.2	86.9	-
Cryptobranchus alleganiensis	47	5961	33	0.6	99.4	81.6	16.06
Notophthalmus viridescens	45.5	5943	42	0.7	99.3	88.6	55.83
Taricha granulosa	45.7	5967	42	0.7	99.3	88.8	58.51
Taricha torosa	45.7	5967	42	0.7	99.3	89.0	36.8
Salamandra salamandra	45.7	5097	237	4.6	95.4	84.1	-
Plethodon cinereus	48.7	5961	48	0.8	99.2	81.8	22.49
Xenopus tropicalis	47	5988	45	0.8	99.2	73.3	-

SCN8A (Na_v1.6)	GC %	Length	Gaps	% Gaps	% Complete	Pairwise % Identity	Average coverage
Ambystoma mexicanum	44.5	5946	30	0.5	99.5	88.0	-
Pleurodeles waltl	44.5	5691	84	1.5	98.5	90.0	-
Cryptobranchus alleganiensis	44.7	5946	21	0.4	99.6	88.2	21.26
Notophthalmus viridescens	44.7	5916	30	0.5	99.5	91.5	49.65
Taricha granulosa	44.8	5952	30	0.5	99.5	91.7	43.78
Taricha torosa	44.8	5907	30	0.5	99.5	91.8	35.75
Salamandra salamandra	44.9	5946	30	0.5	99.5	90.8	-
Plethodon cinereus	44.7	5949	42	0.7	99.3	86.8	32.32
Xenopus tropicalis	41.9	5946	18	0.3	99.7	79.1	-

[a] Number of base pairs in a sequence divided by the total alignment length
[b] Average of all pairwise comparisons within an alignment

Table S2. Synonymous and Nonsynonymous Polymorphism in Salamander Na_v Genes Sequenced for this Study

	SCN1A	*SCN2A*	*SCN3A*	*SCN4A*	*SCN5A*	*SCN8A*
C. alleganiensis **(n=2)**						
Synonymous (P_s)	3	4	1	1	2	0
Nonsynonymous (P_n)	0	2	0	0	0	0
Insertions	0	0	0	0	0	0
Missing exons	0	1	0	0	0	0
P. cinereus **(n=2)**						
Synonymous (P_s)	0	3	3	6	4	0
Nonsynonymous (P_n)	1	0	0	1	0	0
Insertions	0	0	0	0	0	0
Missing exons	0	2	2	1	0	0
N. viridescens **(n=3)**						
Synonymous (P_s)	24	22	35	19	11	12
Nonsynonymous (P_n)	5	2	4	8	1	1
Insertions	1 (39 bases)	0	0	0	0	0
Missing exons	0	2	0	0	0	0
T. torosa **(n=3)**						
Synonymous (P_s)	2	5	2	2	1	0
Nonsynonymous (P_n)	1	2	0	1	1	0
Insertions	0	0	0	1 (3 bases)	0	0
Missing exons	0	2	0	0	0	0
T. granulosa **(n=3)**						
Synonymous (P_s)	4	8	3	10	4	5
Nonsynonymous (P_n)	0	0	0	2	2	0
Insertions	0	0	0	0	0	0
Missing exons	0	2	0	0	0	0

Table S3. Likelihood Ratio Tests from Codeml Site Models

	ℓ	n Parameters	Parameter Estimates	$2\Delta\ell$
***SCN1A* (Na$_v$1.1)**				
Nearly neutral site model (M1a)	-20237.40	18	p_0 = 0.91, (p_1 = 0.09)<br>ω_0 = 0.03, (ω_1 = 1.00)	-
Site selection model (M2a)	-20237.40	20	p_0 = 0.91, p_1 = 0.09, (p_2 = 0.00)<br>ω_0 = 0.03, (ω_1 = 1.00), ω_2 = 50.8	(M2a vs. M1a) **0.00**
Site null model (M7)	-20200.36	18	p = 0.11, q = 1.01	-
Site neutral model (M8a)	-20193.38	19	p = 0.13, q = 1.69<br>p_0 = 0.96, (p_1 = 0.04)	-
Site selection model (M8)	-20193.38	20	p = 0.13, q = 1.69<br>p_0 = 0.96, (p_1 = 0.04), ω_1 = 1.00	(M7 vs. M8) **13.97****<br>(M8 vs. M8a) **0.00**
***SCN2A* (Na$_v$1.2)**				
Nearly neutral site model (M1a)	-21559.11	18	p_0 = 0.89, (p_1 = 0.11)<br>ω_0 = 0.05, (ω_1 = 1.00)	-
Site selection model (M2a)	-21559.11	20	p_0 = 0.89, p_1 = 0.11, (p_2 = 0.00)<br>ω_0 = 0.05, (ω_1 = 1.00), ω_2 = 45.2	(M2a vs. M1a) **0.00**
Site null model (M7)	-21481.17	18	p = 0.19, q = 1.43	-
Site neutral model (M8a)	-21474.94	19	p = 0.22, q = 2.14<br>p_0 = 0.97, (p_1 = 0.03)	-
Site selection model (M8)	-21474.89	20	p = 0.21, q = 2.07<br>p_0 = 0.98, (p_1 = 0.02), ω_1 = 1.09	(M7 vs. M8) **12.56****<br>(M8 vs. M8a) **0.10**
***SCN3A* (Na$_v$1.3)**				
Nearly neutral site model (M1a)	-21487.41	18	p_0 = 0.91, (p_1 = 0.09)<br>ω_0 = 0.03, (ω_1 = 1.00)	-
Site selection model (M2a)	-21487.41	20	p_0 = 0.91, p_1 = 0.09, (p_2 = 0.00)<br>ω_0 = 0.03, (ω_1 = 1.00), ω_2 = 4.73	(M2a vs. M1a) **2.92**
Site null model (M7)	-21439.44	18	p = 0.12, q = 0.99	-
Site neutral model (M8a)	-21406.75	19	p = 0.14, q = 1.84<br>p_0 = 0.96, (p_1 = 0.04)	-
Site selection model (M8)	-21401.10	20	p = 0.13, q = 1.48<br>p_0 = 0.98, (p_1 = 0.02), ω_1 = 1.83	(M7 vs. M8) **76.68****<br>(M8 vs. M8a) **11.30****
***SCN4A* (Na$_v$1.4)**				
Nearly neutral site model (M1a)	-21591.93	18	p_0 = 0.83, (p_1 = 0.17)<br>ω_0 = 0.03, (ω_1 = 1.00)	-
Site selection model (M2a)	-21591.93	20	p_0 = 0.83, p_1 = 0.17, (p_2 = 0.00)<br>ω_0 = 0.03 (ω_1 = 1.00), ω_2 = 107.5	(M2a vs. M1a) **0.00**
Site null model (M7)	-21528.57	18	p = 0.14, q = 0.78	-
Site neutral model (M8a)	-21521.16	19	p = 0.16, q = 1.54<br>p_0 = 0.93, (p_1 = 0.07)	-
Site selection model (M8)	-21521.09	20	p = 0.16, q = 1.27<br>p_0 = 0.94, (p_1 = 0.06), ω_1 = 1.09	(M7 vs. M8) **14.82****<br>(M8 vs. M8a) **0.14**
***SCN5A* (Na$_v$1.5)**				
Nearly neutral site model (M1a)	-22525.65	18	p_0 = 0.88 (p_1 = 0.12)<br>ω_0 = 0.04, (ω_1 = 1.00)	-
Site selection model (M2a)	-22523.34	20	p_0 = 0.88, p_1 = 0.12, (p_2 = 0.00)<br>ω_0 = 0.03 (ω_1 = 1.00), ω_2 = 30.1	(M2a vs. M1a) **4.63**
Site null model (M7)	-22482.16	18	p = 0.15, q = 1.04	-
Site neutral model (M8a)	-22465.19	19	p = 0.20, q = 2.28<br>p_0 = 0.95, (p_1 = 0.05)	-
Site selection model (M8)	-22465.19	20	p = 0.20, q = 2.28<br>p_0 = 0.95, (p_1 = 0.05), ω_1 = 1.00	(M7 vs. M8) **33.94****<br>(M8 vs. M8a) **0.00**

***SCN8A* (Na$_v$1.6)**				
Nearly neutral site model (M1a)	-18903.82	18	p_0 = 0.94 (p_1 = 0.06)<br>ω_0 = 0.02, (ω_1 = 1.00)	-
Site selection model (M2a)	-18903.82	20	p_0 = 0.94, p_1 = 0.06, (p_2 = 0.00)<br>ω_0 = 0.02 (ω_1 = 1.00), ω_2 = 89.1	(M2a vs. M1a) **0.00**
Site null model (M7)	-18911.33	18	p = 0.09, q = 0.99	-
Site neutral model (M8a)	-18879.57	19	p = 0.12, q = 2.25<br>p_0 = 0.97, (p_1 = 0.03)	-
Site selection model (M8)	-18878.56	20	p = 0.11, q = 1.70<br>p_0 = 0.98, (p_1 = 0.02), ω_1 = 1.42	(M7 vs. M8) **65.54****<br>(M8 vs. M8a) **2.02**

** P-value < 0.01, determined by likelihood ratio test using χ^2 distribution

* P-value < 0.05, determined by likelihood ratio test using χ^2 distribution

Table S4. Likelihood Ratio Tests from Codeml Branch and Branch-Site Models Comparing Newts (Foreground) with Other Salamanders (Background)

	ℓ	n Parameters	Parameter Estimates	$2\Delta\ell$
SCN1A **(Na_v1.1)**				
One ratio model (M0)	-20546.55	17	ω = 0.07	**-**
Branch model	-20526.69	18	ω salamanders = 0.07, ω newts = 0.17	(Branch vs. M0) **39.71****
Branch-site neutral model (A1)	-20229.55	19	p_0 = 0.86, p_1 = 0.08, (p_2 = 0.05) ω_0 = 0.03, (ω_1 = 1.00)	-
Branch-site selection model (A)	-20229.55	20	p_0 = 0.86, p_1 = 0.08, (p_2 = 0.05) ω_0 = 0.03, (ω_1 = 1.00), ω_2 = 1.00	(A vs. A1) **0.00**
SCN2A **(Na_v1.2)**				
One ratio model (M0)	-21838.97	17	ω = 0.09	**-**
Branch model	-21838.97	18	ω salamanders = 0.09, ω newts = 0.09	(Branch vs. M0) **0.00**
Branch-site neutral model (A1)	-21558.98	19	p_0 = 0.89, p_1 = 0.11, (p_2 = 0.00) ω_0 = 0.05, (ω_1 = 1.00)	-
Branch-site selection model (A)	-21558.52	20	p_0 = 0.89, p_1 = 0.11, (p_2 = 0.00) ω_0 = 0.05, (ω_1 = 1.00), ω_2 = 7.84	(A vs. A1) **0.94**
SCN3A **(Na_v1.3)**				
One ratio model (M0)	-21867.12	17	ω = 0.08	**-**
Branch model	-21853.31	18	ω salamanders = 0.07, ω newts = 0.15	(Branch vs. M0) **27.63****
Branch-site neutral model (A1)	-21471.92	19	p_0 = 0.89, p_1 = 0.08, (p_2 = 0.03) ω_0 = 0.03, (ω_1 = 1.00)	-
Branch-site selection model (A)	-21468.13	20	p_0 = 0.90, p_1 = 0.09, (p_2 = 0.01) ω_0 = 0.03, (ω_1 = 1.00), ω_2 = 5.50	(A vs. A1) **7.59***
SCN4A **(Na_v1.4)**				
One ratio model (M0)	-22097.03	17	ω = 0.11	**-**
Branch model	-22082.43	18	ω salamanders = 0.10, ω newts = 0.23	(Branch vs. M0) **29.20****
Branch-site neutral model (A1)	-21576.31	19	p_0 = 0.75, p_1 = 0.15, (p_2 = 0.10) ω_0 = 0.03, (ω_1 = 1.00)	-
Branch-site selection model (A)	-21576.16	20	p_0 = 0.78, p_1 = 0.16, (p_2 = 0.06) ω_0 = 0.03, (ω_1 = 1.00), ω_2 = 1.61	(A vs. A1) **0.30**
SCN5A **(Na_v1.5)**				
One ratio model (M0)	-22980.74	17	ω = 0.09	**-**
Branch model	-22978.73	18	ω salamanders = 0.09, ω newts = 0.12	(Branch vs. M0) **4.00**
Branch-site neutral model (A1)	-22525.65	19	p_0 = 0.88, p_1 = 0.11, (p_2 = 0.00) ω_0 = 0.02, (ω_1 = 1.00)	-
Branch-site selection model (A)	-22525.65	20	p_0 = 0.88, p_1 = 0.11, (p_2 = 0.00) ω_0 = 0.02, (ω_1 = 1.00), ω_2 = 66.9	(A vs. A1) **0.00**
SCN8A **(Na_v1.6)**				
One ratio model (M0)	-19187.81	17	ω = 0.06	-
Branch model	-19183.65	18	ω salamanders = 0.05, ω newts = 0.09	(Branch vs. M0) **8.33***
Branch-site neutral model (A1)	-18903.82	19	p_0 = 0.94, p_1 = 0.06, (p_2 = 0.00) ω_0 = 0.02, (ω_1 = 1.00)	-
Branch-site selection model (A)	-18903.82	20	p_0 = 0.94, p_1 = 0.06, (p_2 = 0.00) ω_0 = 0.02, (ω_1 = 1.00), ω_2 = 1.00	(A vs. A1) **0.00**

** P-value < 0.01, determined by likelihood ratio test using χ^2 distribution
* P-value < 0.05, determined by likelihood ratio test using χ^2 distribution

Table S5. Posterior Probabilities for Sites with Elevated ω Values in Toxic Newts

Site[a]	Exon	Nav1.1	Nav1.2	Nav1.3	Nav1.4	Nav1.5	Nav1.6
12	1				0.62		
27	1		0.91	0.55			
38	1				0.52		
120	2					0.77	0.75
155	3	0.53					
249	6	0.64					
278	6	0.58					
338	6			0.56			
340	6	0.79					
452	9			0.61			
460	9			0.60			
493	10	0.54					
543	13			0.98			
549	13			0.55			
621	14			0.59			
719	15			0.58	0.50		
**756**[b]	**15**				**0.94**		
**759**[b]	**15**			**0.61**			
767	15			0.61			
774	15			0.66			
829	16	0.53					
837	16	0.60			0.54		
842	16	0.59			0.51		
843	16			0.51			
845	16	0.60					
877	16	0.51					
879	16			0.57			
881	16	0.60					
884	16			0.97			
887	16			0.60			
898	16			0.59			
911	16	0.61					
921	16	0.51					0.74
936	16	0.53					
940	16					0.69	
946	17	0.60					
957	17	0.58					
960	17	0.59					
965	17	0.95					
968	17	0.53					
981	17				0.53		
993	18				0.52		
1006	18			0.51	0.54		
1028	18				0.97		
1046	19				0.52		
1127	20					0.56	
1179	21	0.59					
1187	21				0.52		
1189	21	0.51					
1191	21			0.86			
1194	21	0.60		0.83			
1224	21	0.61					
**1240**[b]	**22**				**0.53**		
1250	22	0.61					
1254	23			0.54			
1257	23			0.97			
1261	23	0.52					
1262	23	0.61					
1367	25	0.60					
1383	25				0.52		
1390	25				0.55		
**1519**[b]	**26**				**0.52**		
**1529**[b]	**26**	**0.52**					
**1532**[b]	**26**				**0.98**		
1542	26			0.60			
1631	26	0.52					

1737	26		0.56	
1738	26		0.51	
1739	26	0.53		
1741	26		0.51	
1744	26		0.54	
1748	26		0.52	0.53
1752	26		0.83	
1774	26	0.61		
1796	26	0.56		
1817	26	0.59		
1820	26	0.59		
1827	26	0.51		
1832	26	0.52		
1939	26	0.52		

[a] Site numbers are in reference to amino acid positions in the rat $Na_v1.4$ channel (accession number AAA41682)

[b] Known tetrodotoxin binding sites

Table S6. Posterior Probabilities for Sites Under Putative Positive Selection in All Salamanders

Site[a]	Exon	$Na_v1.1$[b]	$Na_v1.2$[b]	$Na_v1.3$[b]	$Na_v1.4$[b]	$Na_v1.5$[b]	$Na_v1.6$[b]
19	1			0.58; 0.81			
22	1						0.70; 0.93
43	1			0.75; 0.95			
46	1		0.51; 0.60				
56	1			0.56; 0.79			
73	1	0.52; 0.63		0.51		0.60; 0.84	0.68; 0.91
74	1						0.76; 0.96
80	1					0.54	
115	2				0.93		
155	3					0.52	
185	4					0.90; 0.99	
202	4			0.56			
209	5					0.62	
287	6	0.65; 0.88	0.51; 0.83				
289	6	0.54					
290	6		0.56; 0.67				
292	6				0.86		
294	6	0.62; 0.85				0.52	
295	6					0.67; 0.93	
297	6				0.82		
298	6	0.56; 0.78				0.51; 0.71	0.60
300	6	0.53; 0.70	0.60; 0.82				
301	6					0.56	
302	6	0.51; 0.62	0.56; 0.68	0.65; 0.88			
306	6		0.71; 0.91		0.67		
307	6			0.68; 0.92		0.53	
309	6			0.59; 0.81		0.60	
311	6				0.56	0.58	
325	6				0.92		
326	6				0.77		
328	6		0.53				
329	6			0.51; 0.66			
330	6					0.53	
332	6						0.54; 0.72
333	6	0.66		0.64; 0.88	0.63		0.56; 0.77
337	6		0.70; 0.90	0.81; 0.98			
338	6	0.55; 0.75					
339	6	0.61; 0.82	0.61; 0.75				0.57; 0.79
340	6			0.62		0.63	0.52; 0.61
344	7	0.60; 0.80	0.64; 0.82	0.51; 0.67		0.55	
345	7					0.69; 0.93	0.52; 0.61
346	7					0.69; 0.94	
348	7			0.70; 0.92		0.66; 0.90	
351	7	0.60; 0.81					
358	7	0.55; 0.71	0.58; 0.72				
365	8			0.59; 0.80		0.62; 0.88	
366	8					0.52; 0.73	
368	8	0.52; 0.70	0.56; 0.71		0.63		0.60; 0.82
374	8						0.62
**401**[c]	**8**	**0.61; 0.85**		**0.51; 0.65**			**0.51; 0.68**
423	9				0.51		
476	10				0.57		
485	10	0.51; 0.64			0.76		
486	10				0.61		
487	10	0.53; 0.71					
492	10				0.65		
505	10				0.55		
521	10				0.79		
555	13	0.58; 0.74	0.53; 0.61	0.78; 0.97			
557	13	0.60		0.68; 0.91	0.87		0.53; 0.62
560	13	0.65					
563	13	0.66; 0.88				0.56	
567	13						0.51
598	13	0.62; 0.87			0.56	0.73; 0.96	0.57; 0.81
601	13			0.80; 0.97			
602	13					0.66; 0.91	0.65; 0.85

606	13		0.52				
609	13					0.53	
654	14			0.76; 0.96			
728	15		0.57; 0.65				
729	15			0.51; 0.64	0.97	0.50; 0.71	0.75; 0.96
732	15		0.52; 0.56	0.61; 0.81	0.78	0.71; 0.94	
739	15					0.54; 0.79	
774	15		0.70; 0.90				
828	16					0.70	
830	16						0.87; 0.99
832	16	0.55	0.57				0.85; 0.99
840	16						0.53; 0.71
841	16				0.73	0.64	
846	16					0.61	
848	16					0.57; 0.82	
849	16						0.51
850	16					0.63	
852	16			0.56; 0.69		0.55; 0.80	
864	16			0.54; 0.66			
877	16					0.68; 0.93	
878	16					0.61	
881	16				0.81	0.51	
886	16					0.52; 0.76	
887	16		0.58				
899	16					0.54	
912	16		0.60; 0.82				
913	16		0.64; 0.82				
916	16				0.50		
943	17				0.55		
950	17					0.64	
951	17			0.73; 0.94			
952	17	0.55					
967	17	0.53; 0.70					
971	17				0.96		
972	17	0.69; 0.91			0.67		
976	17					0.53	
978	17	0.58; 0.79					
979	17			0.86; 0.99			
980	17					0.52; 0.72	
981	17	0.53; 0.71					
985	17						0.64
997	18				0.82		
999	18	0.59					
1003	18					0.61	
1004	18			0.76; 0.96			
1006	18					0.68; 0.92	
1008	18				0.61		
1009	18		0.64; 0.85			0.52; 0.74	0.77; 0.97
1012	18					0.52; 0.75	
1106	20					0.55; 0.79	
1111	20					0.64; 0.88	
1113	20					0.62; 0.85	
1115	20					0.65	
1187	21			0.50; 0.66			
1188	21					0.57; 0.82	
1189	21			0.54; 0.68			0.53
1192	21			0.72; 0.93			
1193	21	0.70; 0.93	0.63; 0.86	0.87; 0.99			0.73; 0.93
1194	21				0.57		
1195	21			0.57; 0.82			
1203	21	0.59; 0.78					0.64; 0.88
1204	21	0.58		0.53			0.69; 0.92
1207	21	0.60; 0.81		0.88; 0.99	0.76	0.61; 0.86	
1208	21			0.60; 0.81			0.55; 0.74
1211	21				0.92		
1216	21					0.58	
1251	22			0.67; 0.88			
1253	23	0.57; 0.80					
1254	23	0.59; 0.81					
1257	23				0.89		

1260	23		0.52				
1332	24	0.64; 0.85	0.53; 0.66				
1334	25		0.65; 0.87				
1351	25				0.67		
1372	25				0.72		0.69; 0.92
1380	25	0.57		0.69			
1390	25					0.62	
1424	25				0.61		
**1533**[c]	**26**	**0.50; 0.67**	**0.55; 0.71**				
1542	26					0.66	
1543	26	0.67; 0.90				0.70	
1549	26	0.50; 0.66		0.66; 0.89			
1550	26				0.89		
1551	26		0.58; 0.78	0.68; 0.91		0.57; 0.80	
1553	26				0.54		
1556	26				0.95		
1558	26				0.95	0.56	
1618	26				0.54		
1623	26		0.56; 0.78				
1631	26			0.53			
1635	26	0.53					
1725	26	0.60				0.58; 0.83	
1726	26		0.62; 0.81				
1733	26	0.62; 0.81					
1736	26				0.53		
1739	26		0.56; 0.70				
1742	26				0.66		
1745	26		0.53; 0.66				
1746	26		0.70; 0.92		0.91		
1747	26		0.62; 0.83		0.81		
1748	26		0.63; 0.84				
1749	26				0.71		
1751	26				0.63		
1752	26				0.61		
1754	26				0.93		
1755	26		0.62; 0.79	0.57			
1757	26						
1760	26				0.74		
1767	26				0.61		
1769	26			0.58	0.61		
1773	26				0.57		
1780	26				0.78		
1782	26				0.60		
1784	26				0.74		
1789	26				0.87		
1798	26				0.69		
1802	26				0.87		
1808	26				0.77		
1809	26				0.51		
1816	26				0.67		
1817	26				0.51		
1819	26				0.69		
1822	26			0.58; 0.82			
1828	26					0.62	
1835	26				0.98		
1839	26				0.58		

[a] Site numbers are in reference to amino acid positions in the rat $Na_v1.4$ channel (accession number AAA41682)

[b] Numbers indicate posterior probabilities of positive selection from empirical Bayes estimates in PAML. A single value indicates detection from the M8 model only and two values indicate detection from both the M2a model and M8 models.

[c] Known tetrodotoxin binding sites

Table S7. Amphibian Na_v Sequences Used for Targeted NGS Probe Design

Species	Source[a]	Best BLAST hit	Accession
Ambystoma mexicanum	WGS NCBI	*SCN1A*	gb|PGSH01113157.1
Ambystoma mexicanum	WGS NCBI	*SCN2A*, *SCN3A*	gb|PGSH01109388.1
Ambystoma mexicanum	WGS NCBI	*SCN4A*	gb|PGSH01101866.1, gb|PGSH01095590.1, gb|JXRH01331098.1
Ambystoma mexicanum	WGS NCBI	*SCN5A*	gb|PGSH01008813.1
Ambystoma mexicanum	WGS NCBI	*SCN8A*	gb|PGSH01049067.1
Hynobius chinensis	TSA NCBI	*SCN1A*	gb|GAQK01012416.1, gb|GAQK01089723.1, gb|GAQK01123640.1, gb|GAQK01022956.1, gb|GAQK01037701.1, gb|GAQK01110837.1, gb|GAQK01035933.1, gb|GAQK01049457.1, gb|GAQK01026323.1 gb|GAQK01118202.1, gb|GAQK01062486.1, gb|GAQK01026323.1, gb|GAQK01062486.1
Hynobius chinensis	TSA NCBI	*SCN2A*	gb|GAQK01012415.1, gb|GAQK01089724.1, gb|GAQK01123639.1, gb|GAQK01062305.1, gb|GAQK01047585.1, gb|GAQK01096581.1, gb|GAQK01044980.1, gb|GAQK01086119.1, gb|GAQK01096592.1, gb|GAQK01106518.1, gb|GAQK01122588.1
Hynobius chinensis	TSA NCBI	*SCN4A*	gb|GAQK01140156.1, gb|GAQK01024534.1, gb|GAQK01021831.1, gb|GAQK01021830.1, gb|GAQK01082803.1, gb|GAQK01071419.1, gb|GAQK01071419.1
Hynobius chinensis	TSA NCBI	*SCN5A*	gb|GAQK01128205.1, gb|GAQK01083790.1, gb|GAQK01027263.1, gb|GAQK01027805.1, gb|GAQK01015614.1, gb|GAQK01012146.1, gb|GAQK01014539.1, gb|GAQK01014539.1
Hynobius chinensis	TSA NCBI	*SCN8A*	gb|GAQK01067521.1, gb|GAQK01020756.1, gb|GAQK01038507.1, gb|GAQK01045113.1, gb|GAQK01022217.1, gb|GAQK01096591.1, gb|GAQK01071418.1, gb|GAQK01071418.1
Hynobius retardatus	TSA NCBI	*SCN1A*	gb|LE210884.1, gb|LE107081.1, gb|LE105972.1
Hynobius retardatus	TSA NCBI	*SCN3A*	gb|LE175129.1
Hynobius retardatus	TSA NCBI	*SCN4A*	gb|LE175126.1, gb|LE175128.1
Hynobius retardatus	TSA NCBI	*SCN5A*	gb|LE143587.1, gb|LE143588.1
Hynobius retardatus	TSA NCBI	*SCN8A*	gb|LE175125.1

Lyciasalamandra atifi	Transcriptome assembly provided by Miguel Vences (Rodríguez et al. 2017)	-	-
Nanorana parkeri	WGS NCBI	*SCN1A, SCN2A, SCN3A*	gb|NW_017306417.1
Nanorana parkeri	WGS NCBI	*SCN4A*	gb|NW_017306748.1
Nanorana parkeri	WGS NCBI	*SCN5A*	gb|NW_017306389.1
Nanorana parkeri	WGS NCBI	*SCN8A*	gb|NW_017307114.1
Notophthalmus viridescens	http://sandberg.cmb.ki.se/redspottednewt/	-	-
Paramesotriton hongkonginensis	Sequence Read Archive NCBI SRX796492	-	-
Pleurodeles waltl	Whole genome assembly provided by Ahmed Elewa (Elewa et al. 2017)	*SCN1A*	abyss_v4.2_66066951
Pleurodeles waltl	Transcriptome assembly from iNewt Database: http://www.nibb.ac.jp/imori/main/	*SCN1A*	TRINITY_DN288824_c1_g3_i7
Pleurodeles waltl	Whole genome assembly provided by Ahmed Elewa (Elewa et al. 2017)	*SCN2A*	abyss_v4.2_66112789
Pleurodeles waltl	Whole genome assembly provided by Ahmed Elewa (Elewa et al. 2017)	*SCN3A*	abyss_v4.2_66060341
Pleurodeles waltl	Transcriptome assembly from iNewt Database: http://www.nibb.ac.jp/imori/main/	*SCN3A*	TRINITY_DN288824_c1_g3_i6
Pleurodeles waltl	Whole genome assembly provided by Ahmed Elewa (Elewa et al. 2017)	*SCN4A*	abyss_v4.2_48183054
Pleurodeles waltl	Transcriptome assembly from iNewt Database: http://www.nibb.ac.jp/imori/main/	*SCN4A*	TRINITY_DN288824_c1_g2_i5
Pleurodeles waltl	Whole genome assembly provided by Ahmed Elewa (Elewa et al. 2017)	*SCN5A*	abyss_v4.2_66164693
Pleurodeles waltl	Whole genome assembly provided by Ahmed Elewa (Elewa et al. 2017)	*SCN8A*	abyss_v4.2_66123907
Salamandra atra	Transcriptome assembly provided by Miguel Vences (Rodríguez et al. 2017)	-	-
Salamandra infraimmaculata	Transcriptome assembly provided by Miguel Vences (Rodríguez et al. 2017)	-	-
Salamandra salamandra	TSA NCBI	*SCN1A*	gb|GIKK01030996.1, gb|GIKK01027377.1, gb|GIKK01026170.1, gb|GIKK01027688.1
Salamandra salamandra	TSA NCBI	*SCN2A*	gb|GIKK01012950.1, gb|GIKK01007670.1, gb|GIKK01006682.1
Salamandra salamandra	TSA NCBI	*SCN3A*	gb|GIKK01031859.1
Salamandra salamandra	TSA NCBI	*SCN4A*	gb|GIKK01007017.1, gb|GIKK01015583.1
Salamandra salamandra	TSA NCBI	*SCN5A*	gb|GIKK01011042.1
Salamandra salamandra	TSA NCBI	*SCN8A*	gb|GIKK01019313.1, gb|GIKK01023854.1, gb|GIKK01002548.1
Tylototriton wenxianensis	TSA NCBI	*SCN4A*	gb|GESS01000732.1, gb|GESS01024789.1, gb|GESS01029581.1, gb|GESS01063809.1

Tylototriton wenxianensis	TSA NCBI	*SCN5A*	gb|GESS01016882.1, gb|GESS01035197.1
Xenopus tropicalis	WGS NCBI	*SCN1A*	gb|AAMC03035440.1
Xenopus tropicalis	WGS NCBI	*SCN2A*	gb|AAMC03035445.1
Xenopus tropicalis	WGS NCBI	*SCN3A*	gb|AAMC03035458.1, gb|AAMC03035459.1, gb|AAMC03035460.1
Xenopus tropicalis	WGS NCBI	*SCN4A*	gb|AAMC03036452.1
Xenopus tropicalis	WGS NCBI	*SCN5A*	gb|AAMC03022243.1, gb|AAMC03022245.1, gb|AAMC03022246.1, gb|AAMC03022247.1, gb|AAMC03022249.1, gb|AAMC03022250.1, gb|AAMC03022251.1
Xenopus tropicalis	WGS NCBI	*SCN8A*	gb|AAMC03008918.1, gb|AAMC03008917.1, gb|AAMC03008916.1

[a] WGS – whole genome shotgun database, TSA – transcriptome shotgun assembly database
